# Protective Effect of *Gochujang* on Inflammation in a DSS-Induced Colitis Rat Model

**DOI:** 10.3390/foods10051072

**Published:** 2021-05-12

**Authors:** Patience Mahoro, Hye-Jung Moon, Hee-Jong Yang, Kyung-Ah Kim, Youn-Soo Cha

**Affiliations:** 1 Department of Food Science and Human Nutrition & Obesity Research Center, Jeonbuk National University, Jeonju 54896, Korea; mahoropapy10@gmail.com (P.M.); moonhj2018@daum.net (H.-J.M.); 2 Microbial Institute for Fermentation Industry (MIFI), Sunchang 56048, Korea; godfiltss@naver.com; 3 Department of Food and Nutrition, Chungnam National University, Daejeon 34134, Korea; kakim@cnu.ac.kr; 4 Obesity Research Center, Jeonbuk National University, Jeonju 54896, Korea

**Keywords:** *Gochujang*, dextran sulfate sodium (DSS), anti-inflammation, microbiota

## Abstract

*Gochujang* is a traditional Korean fermented soy-based spicy paste made of *meju* (fermented soybean), red pepper powder, glutinous rice, and salt. This study investigated the anti-inflammatory effects of *Gochujang* containing salt in DSS-induced colitis. Sprague–Dawley (SD) rats were partitioned into five groups: normal control, DSS control, DSS + salt, DSS + mesalamine, and DSS + *Gochujang* groups. They were tested for 14 days. *Gochujang* improved the disease activity index (DAI), colon weight/length ratio, and colon histomorphology, with outcomes similar to results of mesalamine administration. Moreover, *Gochujang* decreased the serum levels of IL-1β and IL-6 and inhibited TNF-α, IL-6, and IL-1β mRNA expression in the colon. *Gochujang* downregulated the expression of iNOS and COX-2 and decreased the activation of NF-κB in the colon. *Gochujang* induced significant modulation in gut microbiota by significantly increasing the number of *Akkermansia muciniphila* while decreasing the numbers of *Enterococcus faecalis* and *Staphylococcus sciuri*. However, compared with the DSS group, the salt group did not significantly change the symptoms of colitis or cytokine levels in serum and colon. Moreover, the salt group significantly decreased the gut microflora diversity. *Gochujang* mitigated DSS-induced colitis in rats by modulating inflammatory factors and the composition of gut microflora, unlike the intake of salt alone.

## 1. Introduction

Inflammatory bowel disease (IBD) comprises bowel abnormalities that cause chronic inflammation of the gastrointestinal (GI) tract. Crohn’s disease (CD) and ulcerative colitis (UC) are the two major forms of IBD and are marked by periods of quiescence or activity [1]. Both forms are life-threatening and are among the three top factors that contribute to colorectal cancer. However, UC is more frequently reported as a causal agent of colorectal cancer than is CD [2]. Management of this relapsing-remitting disorder includes corticosteroids, immunomodulators, and amino salicylates, such as mesalamine, a targeting remission of inflammation along with maintenance of the quiescent state of the disease [3,4]. Healthy dietary habits play a key role in alleviating UC symptoms [5,6]. Further, several studies have shown that fermented foods have a positive effect on intestinal health by influencing the profile and function of gut microbiota [7,8,9].

In contrast, constant exposure to a high level of salt (NaCl) in the diet modulates gut microbiota composition and function by promoting a predisposition to disease through a pro-inflammatory state in the gut. Research revealed that feeding mice a salt-enriched diet worsens experimentally-induced colitis [10]. A high salt diet (HSD) helps to sustain the pro-inflammatory microbiota by weakening fecal microflora activity and structure in the colonic community, as well as by suppressing mucosal immunity of the upper part of intestine, which in turn induces expression of pro-inflammatory genes and promotes inflammatory disorders [11].

*Gochujang*, a traditional Korean fermented condiment, consists of glutinous rice, *meju* (fermented soybean) powder, red pepper powder, and a large amount of salt [12]. *Gochujang* as a fermented food has been suggested to help reduce the risk of several health conditions (due to its anti-cancer properties [13]), insulin resistance [14], anti-obesity properties [15], and antioxidant capacity [16]. *Gochujang* contains 5~11% salt, but its main bioactive compounds have been identified as capsaicin of red pepper powder, isoflavones of *meju*, and probiotics [12,17,18]. According to reports, these components help in alleviating colitis symptoms through their anti-inflammatory activities [19,20,21,22,23,24,25]. However, there is a lack of research on reducing signs and symptoms of colitis with *Gochujang*.

Therefore, we hypothesized that *Gochujang* could exert an anti-inflammatory effect. This experiment evaluated the potential protective functions of *Gochujang* containing high salt against dextran sodium sulfate (DSS)-induced colitis in Sprague–Dawley rats (SD rats).

## 2. Materials and Methods

### 2.1. Animal Study

All animal-related processes were performed in accordance with an experimental plan endorsed by the Animal Ethics Committee of Jeonbuk National University (CBNU 2020-073). SD rats (5 weeks old, male, 200–220 g body weight) were ordered from DooYeol Biotech (Seoul, Korea) to be housed under a light-controlled cycle (12 h), at 23 ± 2 °C and with humidity regulated at 55 ± 5%. They were allowed unrestricted access to food (AIN-93 laboratory diet) and drinking water. *Gochujang* was provided by the Microbial Institute for Fermentation Industry (Sunchang-gun, Jeollabuk-do, Korea).

Following a one-week acclimatization period, rats were used in a 14-day experiment, beginning with a random split into five groups (*n* = 7): normal control (NOR), DSS control (DSS), DSS + salt (SAL), DSS + mesalamine (MES), and DSS + *Gochujang* (GCJ). Colitis was induced during 7 days in DSS, SAL, MES, and GCJ groups through free access to drinking water with a DSS concentration of 3% (DSS molecular weight: 36–50 kDa, MP Biomedicals, Irvine, CA, USA); followed by a 7-day recovery phase of drinking water without DSS until sacrifice. All experimental solutions were administered orally throughout the duration of the whole 14-day experiment (Figure S1). The normal and DSS groups received vehicle (distilled water), the SAL group received salt at 348.6 mg/kg body weight (BW), the MES group received mesalamine at 3.7 mg/kg BW, and the GCJ group received *Gochujang* at 2 g/kg BW. *Gochujang*’s salt concentration was determined as 8.72% using Mohr’s method [26], in which the same amount of salt was dissolved in distilled water at 4% NaCl (*w*/*v*) and administered based on references regarding the dose of salt in the induction of colitis [10,11,27]. In previous studies, the administration of mesalamine (7.4 mg/kg BW) to mice was shown to prevent colitis [28]. Mesalamine was then administered according to the corresponding surface area dose conversion formula [29].

### 2.2. Disease Activity Index (DAI) and Sample Collection

In order to assess the severity of acute colitis symptoms, the disease activity index (DAI) was evaluated daily for body weight loss, stool consistency, and bloody stool. On the last day, rats were euthanized and colons were excised from the caeco-colonic junction to the rectum. Excised colons were measured for length and weight after the stool was removed. Part of the colon was fixed in 10% buffered formalin solution for histological analysis. The remaining colon tissue was opened longitudinally, frozen in liquid nitrogen, and stored at −70 °C.

### 2.3. Histological Analysis

Staining of colon tissues fixed in 10% formalin was performed in the Korean Pathology Technical Center (KP&T, Cheongju, Korea). In brief, after being embedded in paraffin, the tissues were excised into 5-mm-thick sections and then dyed using hematoxylin and eosin (H&E). The samples were analyzed at ×100 magnification by Axiophot Zeiss ZI microscope (Carl Zeiss, Gottingen, Germany) installed in the Center for University-wide Research Facilities (CURF) at Jeonbuk National University.

### 2.4. Enzyme-Linked Immunosorbent Assay (ELISA)

The serum concentrations of inflammatory-associated cytokines such as tumor necrosis factor-alpha (TNF-α), IL-6, and IL-1β were assessed via commercial ELISA kits (R&D Systems, Minneapolis, MN, USA) according to the manufacturer’s instructions.

### 2.5. Quantitative Real-Time PCR (qRT-PCR) Analysis

The expression of the roles of inflammation-related genes TNF-α, IL-1β, and IL-6 in the colonic tissue was determined by a qRT-PCR assay. Total RNA was extracted via the QIAGEN RNA easy mini kit (QIAGEN GmbH, Hilden, Germany), followed by the determination of purity. Further, cDNA was generated using a cDNA reverse transcription kit (TaKaRa Bio, Otsu, Japan), and real-time PCR was executed using a SYBR Green PCR Master Mix (Toyobo, Osaka, Japan). The primer sets used in this study are listed in Table 1.

### 2.6. Western Blot Analysis

Immunoblot analysis was performed to determine the relative expression of phosphoprotein p65 (p-p65), inducible nitric oxide synthase (iNOS), and cyclooxygenase-2 (COX-2) in the colon mucosa. Protein extract was derived from colon tissue emulsified in RIPA lysis buffer (Pierce-Thermo Fisher Scientific, Rockford, MD, USA) mixed with a 1% protease inhibitor and 1% phosphatase inhibitor cocktail (Roche Diagnostics GmbH, Mannheim, Germany). After separating the protein using SDS-polyacrylamide gel electrophoresis, it was transferred onto polyvinylidene difluoride membranes (Bio-Rad Laboratories, Hercules, CA, USA). After the membrane was blocked in 5% skim milk, it was washed with phosphate buffered saline with 0.1% tween 20 (PBS-T). Primary antibodies for p-p65, p65, iNOS, COX-2, and β-actin were purchased from Cell Signaling Technology (Beverly, MA, USA). These were applied overnight at 4 °C and washed with PBS-T. After reacting for 45 min at room temperature with a secondary antibody (Santa Cruz Biotechnologies Inc., Santa Cruz, CA, USA), they were washed with PBS-T, followed by measurement of p-p65, p65, iNOS, COX-2, and β actin expression supported by a ChemiDoc MP imaging system (Bio-Rad, Hercules, CA, USA).

### 2.7. Gut Microbiota Analysis

Fecal samples were frozen in liquid nitrogen and stored at −80 °C until further analyzed. Bacterial genomic DNA from feces was extracted via the Fast DNA Spin kit for soil (MP Bio Laboratories, USA). For amplicon sequencing, PCR reactions were performed targeting the V1, V2, and V3 polymorphic regions of the 16S rRNA genes using the appropriate primer with a barcode. The PCR product was confirmed by 2% agarose gel electrophoresis, and impurities other than the amplification product were removed using a QIAquik PCR purification kit (Qiagen, Valencia, CA, USA). The same amount of PCR product was collected and purified again using the AMPure bead kit (Agencourt Bioscience, Beverly, MA, USA). Then, the length and concentration of the amplified products were confirmed with Bioanalyzer 2100 (Agilent Technologies, Santa Clara, CA, USA) using a DNA 7500 chip. The final amplification was performed by Macrogen Co., Ltd. (Seoul, Korea) according to the manufacturer’s manual using the GS Junior Titanium system (Roche, Germany) sequencer.

### 2.8. Statistics and Analysis

To analyze statistical differences, data were compared using one-way ANOVA in SPSS (version 17.0; SPSS, Inc., Chicago, IL, USA). The significant differences among the groups were analyzed by the Duncan’s multiple range test (*p* < 0.05). Data are expressed as the mean ± standard deviation (SD).

## 3. Results

### 3.1. Effect of Gochujang on UC Symptoms

**Figure 1:**
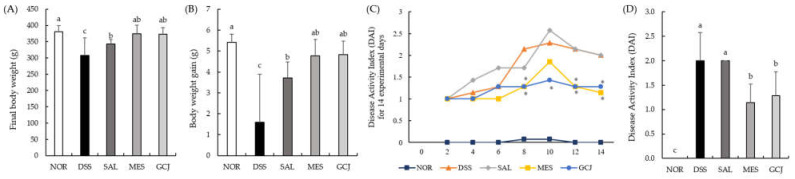
Effects of *Gochujang* on colitis symptoms in DSS-treated rats. (**A**) Final body weight; (**B**) weight gain; (**C**) DAI scores calculated with time (* *p* < 0.05 vs. DSS group); (**D**) DAI scores among the groups at day 14. Data are expressed as the mean ± SD (*n* = 7 per group). Values with different superscripts (a, b, c) are significantly different among groups according to ANOVA with Duncan’s multiple range test at *p* < 0.05. NOR—normal group; DSS—DSS group; SAL—SALT group; MES—mesalamine; GCJ—*Gochujang*.

UC symptoms were evaluated based on factors such as body weight, DAI, and colon-related features. For final body weight, the DSS and SAL groups showed significantly lower body weights than the NOR group, while weights in both the GCJ and MES groups were significantly higher than that of the DSS group (Figure 1A). As shown in Figure 1B, the DSS group was significantly lower in weight gain compared with the other groups (*p* < 0.05). The DAI score was highest on the 8th day in the DSS group and on the 10th day in the SAL group. On the 14th day, the last day of the experiment, the MES and GCJ groups showed significantly lower DAI scores compared with the DSS and SAL groups (*p* < 0.05) (Figure 1C,D).

### 3.2. Effect of Gochujang on Colon Damage

**Figure 2:**
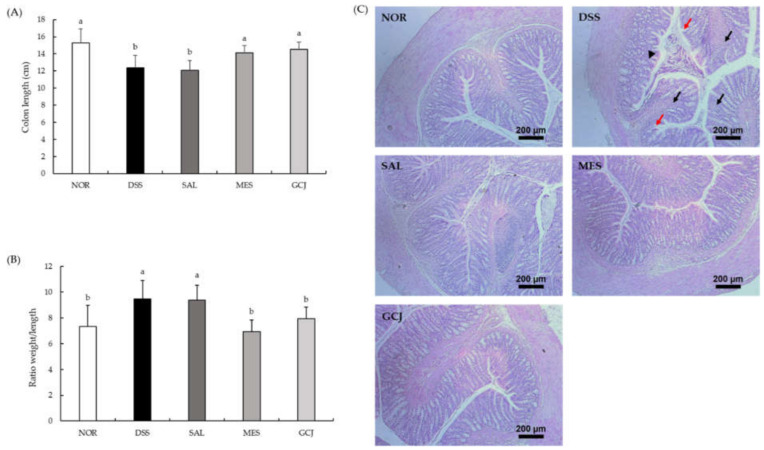
Effects of *Gochujang* on colon tissue damage in DSS-treated rats. (**A**) colon length; (**B**) colon weight/ length ratio; (**C**) H&E staining of the colon tissues (black arrow—inflammatory cell infiltration; red arrow—epithelial erosion; black arrowhead—crypt loss). Data are expressed as the mean ± SD (*n* = 7 per group). Values with different superscripts (a, b) are significantly different among groups according to ANOVA with Duncan’s multiple range test at *p* < 0.05.

We evaluated the reduction in colon length and colon weight/length ratio, as well as other indicators of colorectal inflammation (Figure 2A,B). Colon length was significantly shorter in the DSS and SAL groups compared with the NOR group (*p* < 0.05). However, colons from MES and GCJ groups were significantly longer than those of the DSS and SAL groups (*p* < 0.05). In addition, the colon weight/length ratio was significantly lower in MES and GCJ groups than in DSS and SAL groups (*p* < 0.05). Therefore, *Gochujang* produced alleviation of the symptoms of DSS-induced colitis.

The pathological changes in colon tissue were confirmed by H&E staining (Figure 2C). The DSS and SAL groups indicated crypt disruption of epithelial cells, infiltration of inflammatory cells into mucosa, and loss of goblet cells in the colon. Orally administered mesalamine and *Gochujang* pre-treatments lowered the histopathological alterations induced by DSS, including decreasing inflammatory cell infiltration, goblet cell depletion, and crypt destruction. This resulted in lesser epithelial changes compared with the DSS and SAL groups. Altogether, these results demonstrated that *Gochujang* had a preventive effect against colon tissue damage.

### 3.3. Effect of Gochujang on Serum Levels of Pro-Inflammatory Cytokine

To investigate the anti-inflammatory effect of *Gochujang*, serum levels of TNF-α, IL-6, and IL-1β were measured (Table 2). The levels of IL-6 and IL-1β in serum were significantly increased in the DSS and SAL groups compared with the NOR group (*p* < 0.05). However, MES and GCJ groups showed significantly decreased IL-6 and IL-1β levels compared with DSS and SAL groups. Therefore, *Gochujang* showed inhibition of pro-inflammatory cytokines in serum of colitis rats.

### 3.4. Effects of Gochujang on Colonic Inflammation-Related Gene and Protein Expression

**Figure 3:**
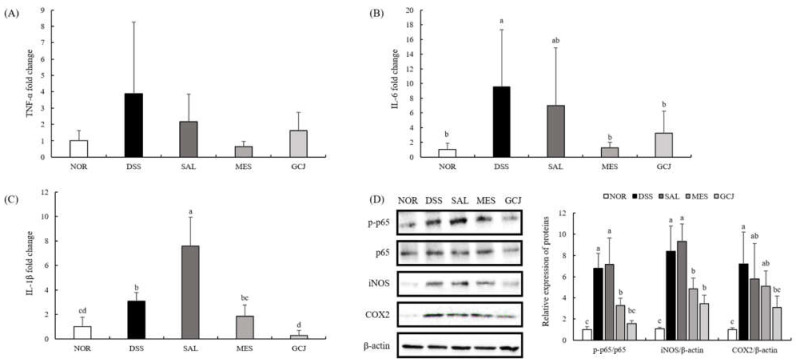
Effects of *Gochujang* on immune response in colon tissue of DSS-treated rats. (**A**–**C**) mRNA expression of colonic pro-inflammatory cytokines (TNF-a, IL-6, and IL-1β); (**D**) expression of inflammatory proteins. Data are expressed as the mean ± SD (*n* = 7 per group). Values with different superscripts (a, b, c, d) are significantly different among groups according to ANOVA with Duncan’s multiple range test at *p* < 0.05.

The mRNA expression of pro-inflammatory genes TNF-α, IL-6, and IL-1β in colon tissue was analyzed using q-PCR (Figure 3A–C). DSS and SAL treatments significantly upregulated mRNA expression of these inflammation-related genes over that of the NOR group (*p* < 0.05). Compared with the DSS and SAL groups, the *Gochujang* and MES groups had lower colonic mRNA expression of TNF-α, IL-6, and IL-1β. Interestingly, the SAL group exhibited the highest mRNA expression of IL-1β, whereas *Gochujang* administration downregulated IL-1β level more effectively (*p* < 0.05) than the MES group.

The iNOS and COX-2 contributed to the severity of intestinal damage by triggering excessive production of inflammatory mediators such as transcription factor NF-κB. We studied their protein expression using immunoblotting analyses (Figure 3D). The expression of p-p65 in colon tissue increased in the DSS group compared with the NOR group, whereas the *Gochujang* group showed decreased level of p-p65 compared with the DSS group (*p* < 0.05). In addition, the expression of iNOS and COX-2 was downregulated in GCJ and MES groups compared with the DSS group (*p* < 0.05).

### 3.5. Effect of Gochujang on Gut Microbiota Dysbiosis

**Figure 4:**
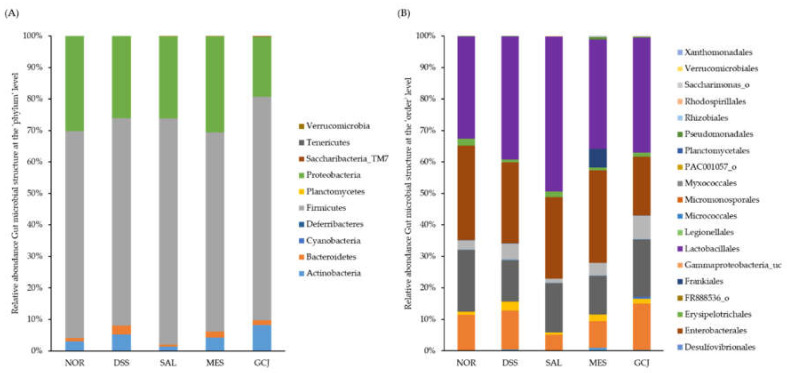
Effects of *Gochujang* on relative abundances of gut microbiota in DSS-treated rats at (**A**) phylum level and (**B**) order level (*n* = 7 per group).

As gut microbiota dysbiosis is associated with colitis, we analyzed the microorganisms in stools to investigate whether *Gochujang* alters gut microbial composition. To evaluate the abundance and diversity of gut microbiota, the alpha-diversity indices OUT, ACE, Chao, Shannon, Simpson, and phylogenetic diversity were analyzed (Table 3). Although there was no significant difference between the DSS-treated group and the NOR group, the SAL group showed a significant decrease in diversity in all indices compared with the DSS group (*p* < 0.05). At the phylum level, the major bacteria analyzed in feces were *Firmicutes*, *Proteobacteria*, *Actinobacteria*, and *Bacteroidetes* (Figure 4A). The DSS group showed no significant difference compared with the NOR group, but the abundance of *Bacteroidetes* tended to increase in the DSS group. At the order level, there was no significant difference in all groups compared with the NOR group. However, *Lactobacillales* tended to be increased in all groups treated with DSS, especially the SAL group (Figure 4B). At the species level, the DSS group had significantly higher *Enterococcus faecalis* and *Staphylococcus sciuri*, but there was no significant difference in *Akkermansia muciniphila* compared with the NOR group. Compared with the DSS group, the GCJ group showed significantly decreased *E. faecalis* and *S. sciuri* but increased *A. muciniphila*. There was no significant difference in the composition of *E. faecium* and *L. reuteri* in GCJ group compared with DSS group, but there was a tendency to increase. The SAL group showed no significant difference in *E. faecalis*, *A. muciniphila*, *E. coli*, or *L. reuteri* compared with the DSS group (Table 4).

## 4. Discussion

*Gochujang* is a traditional Korean dietary spice paste made from fermentation of *meju* (fermented soybean powder), glutinous rice, red pepper powder, malts, and salt. The synergy of fermented soy products and red pepper in *Gochujang* has been reported to have health benefits [30]. Moreover, *Gochujang* has been ranked in the Korean top-15 most commonly consumed foods and top-2 most appreciated condiment seasonings after *kanjang* (fermented soy sauce) [31]. High salt intake is discussed as being associated with activation of pro-inflammatory factors in gastric precancerous lesions such as atrophic gastritis, intestinal metaplasia, gastrointestinal cancer, and colitis [27,32]. However, a previous study has reported that *kanjang* (a Korean fermented soy sauce), though containing a large amount of salt, mitigates DSS-induced colitis. It is suggested that the fermented soy sauce has anti-inflammatory effects compared to salt alone [8]. Therefore, we investigated the anti-inflammatory effect of *Gochujang*, a fermented soybean product containing high salt, in DSS-induced colitis.

The DSS-induced rodent colitis has been reported as a condition that is morphologically and symptomatically similar to UC conditions seen in human clinical symptoms, such as weight reduction, shortening of colon, bloody stool, and diarrhea, which were assessed by DAI score [33,34]. UC also causes histological changes in colon tissues, such as inflammatory cell infiltrate, epithelial damage, and mucosal disruption [35]. In our study, rats treated with DSS revealed greater weight loss, shorter colon, increased colon tissue damage, and higher DAI score compared with NOR rats. Compared with the DSS treatment group, administration of *Gochujang* inhibited the clinical symptoms and the epithelial and mucosal damage of colon tissue. *Gochujang* showed a DAI score similar to that of the positive control group treated with mesalamine. These results suggest that *Gochujang* effectively inhibits symptoms of colitis. Interestingly, rats treated with salt exhibited a high DAI score similar to those treated with DSS alone. These results were consistent with previous studies in that high-salt diet worsened UC pathogenesis [10,36]. Previous studies described that inflammation of the colon increases the activity of inflammation-related cytokines including TNF-α, IL-1β, and IL-6 in serum and colon tissues in murine colitis [20,24,37]. We analyzed serum levels and colonic gene expression to identify changes in TNF-α, IL-6, and IL-1β. The serum levels of IL-6 and IL-1β were significantly lower in the GCJ group than in the DSS and SAL groups.

The gene expression of TNF-α, IL-6, and IL-1β was reduced in the GCJ group compared to the DSS group. In other colitis mouse models, TNF-α, IL-6, and IL-1β in serum and colon were reduced after administering *doenjang*, fermented soybean paste, and isoflavones such as daidzein and genistein, which are bioactive compounds of soybeans [9,20,21,38,39]. Some of the bioactive compounds of *Gochujang* made from *meju* (fermented soybean) are daidzein and genistein [12,16]. These compounds seem to have reduced pro-inflammatory cytokines [40]. However, the salt group had significantly higher IL-1β gene expression in colon tissues than the DSS group. In previous studies on salt and colitis in mice, high salt showed an increased expression of IL-1β and TNF-α in the colon [10,27]. Therefore, in this colitis model, *Gochujang* was found to lessen pro-inflammatory markers when compared to salt alone. It has been reported that increased pro-inflammatory cytokines in mucosal epithelial cells with colitis activate the NF-κB signaling pathway [41]. NF-κB is known to regulate the expression of the inflammatory genes iNOS and COX-2. It is known that p65 is a subunit of NF-κB and is phosphorylated to activate the NF-κB signaling pathway [42]. In this study, the expression of inflammation-related proteins p-p65, iNOS, and COX-2 was examined to confirm the inhibitory pathway for colitis. These results showed downregulation of the expression of iNOS and COX-2, reducing the activation of NF-κB in the GCJ group compared with the DSS group. Similar to our study, *kanjang* showed anti-inflammatory effects by downregulating the expression of iNOS and COX-2 in the colon mucosa of colitis models [8]. In addition, in a rat model of trinitrobenzene sulfonic acid (TNBS)-induced colitis (genistein, a soybean isoflavone) was revealed to be an anti-inflammatory compound with an inhibitory effect on COX-2 expression [43]. Therefore, our findings indicate that *Gochujang* attenuated colon inflammatory responses by inhibiting the NF-κB signaling pathway.

Recently, the link involving gut microbiota and human diseases has received attention in the scientific literature [44]. Drastic changes in gut microbiota of humans with UC were found to be similar to those seen in rat models of colitis induced by DSS [34]. Moreover, gut microbiota dysbiosis is caused by genetic and environmental factors, which is related to colitis [45]. In this study, we identified the effect of *Gochujang* on the gut bacterial composition. Compared with the DSS group, the alpha-diversity index was significantly reduced in the SAL group. A previous study reported that high-salt diets exacerbate colitis and alter gut microbiota in DSS and dinitrobenzene sulfonic acid (DNBS)-induced colitis in mice [11]. Our results also revealed that high salt intake reduced the diversity of gut microflora. However, *Gochujang* containing the same amount of salt did not significantly change the diversity of gut microflora. At the phylum level, the DSS group increased in abundance of *Bacteroidetes* compared with the NOR group. Increased *Bacteroidetes* has been reported as a risk factor for colitis, and our results were in agreement with previous studies [46,47]. The abundance of *E. faecalis* was significantly increased in the DSS group compared with the NOR group. In addition, the abundance of *S. sciuri* was significantly increased in the DSS group. *E. faecalis* showed relatively high abundance in the intestine of IL-10-deficient mice and IBD patients and was reported to be associated with colitis induction [48,49]. *S. sciuri* was found in the colon tissues of patients with colitis and was reported as an oral bacterium that promoted intestinal inflammation in colitis [50,51]. Compared with the DSS group, the GCJ group had significantly reduced abundance of *E. faecalis* and *S. sciuri*, and increased abundance of *A. muciniphila* and *L. reuteri*, which are known to improve intestinal barrier function and inflammatory response [24,52]. Also, the intake of *doenjang* was found to increase *A. muciniphila* in the TNBS-induced colitis model, and our data were similar [9]. Therefore, it was found that *Gochujang*, unlike the equivalent dose of salt alone, improved the abundance of *E. faecalis*, *A. muciniphila*, and *L. reuteri* in the colitis model. Genistein and daidzein, the isoflavones of soybeans, have been reported to increase the relative abundance of *A. muciniphila* in mice and also to improve the composition and diversity of intestinal flora [53,54]. Therefore, the change in the composition of the gut flora seems to be caused by the isoflavones contained in *Gochujang*.

## 5. Conclusions

In this study, the intake of *Gochujang* in DSS-induced colitis rats showed anti-inflammatory effects through down-regulation of inflammatory cytokines and suppression of NF-κB signaling pathways in colon, similar to the effects of mesalamine. *Gochujang* also inhibited the growth of *E. faecalis* and *S. sciuri*, which were increased in the UC, and promoted the growth of *A. muciniphila* and *L. reuteri*, which are beneficial bacteria. However, salt by itself was found to worsen DSS-induced colitis and reduce the diversity of intestinal flora. These results demonstrated that *Gochujang*, a fermented food containing a large amount of salt, has a preventive effect on UC unlike the intake of salt alone. Furthermore, additional clinical trials are considered necessary on the preventive effects of *Gochujang* against colitis.

## Figures and Tables

**Table 1:** Primers used in this study.

Gene Name	Primers	Sequence 5′ to 3′
β-actin	Forward	CCCGCGAGTACAACCTTCT
	Reverse	CGTCATCCATGGCGAACT
TNF-α ^1^	Forward	CCCTGGTACTAACTCCCAGAAA
	Reverse	TGTATGAGAGGGACGGAACC
IL-1β	Forward	CACCTCTCAAGCAGAGCACAG
	Reverse	GGGTTCCATGGTGAAGTCAAC
IL-6	Forward	TCCTACCCCAACTTCCAATGCTC
	Reverse	TTGGATGGTCTTGGTCCTTAGCC

^1^ TNF-α, tumor necrosis factor-alpha; IL-1β, interleukin-1 beta; IL-6, interleukin-6.

**Table 2:** Effects of *Gochujang* on serum levels of inflammatory cytokines in DSS-treated rats.

	TNF-α (pg/mL)	IL-6 (pg/mL)	IL-1β (pg/mL)
NOR	49.97 ± 0.21 ^a^	238.52 ± 3.42 ^c^	43.76 ± 1.02 ^b^
DSS	50.47 ± 0.22 ^a^	251.52 ± 1.83 ^a^	55.66 ± 9.76 ^a^
SAL	50.42 ± 0.24 ^a^	251.69 ± 1.56 ^a^	55.93 ± 11.68 ^a^
MES	47.12 ± 1.93 ^b^	248.87 ± 1.14 ^b^	45.63 ± 1.16 ^b^
GCJ	49.89 ± 0.11 ^a^	247.73 ± 1.39 ^b^	44.81 ± 1.62 ^b^

Data are expressed as the mean ± SD (*n* = 7 per group). Values with different superscripts (a, b, c) are significantly different among groups according to ANOVA with Duncan’s multiple range test at *p* < 0.05. NOR, normal group; DSS, DSS group; SAL, SALT group; MES, Mesalamine; GCJ, *Gochujang*.

**Table 3:** Effects of *Gochujang* on diversity indexes of the gut microbiota in DSS-treated rats.

	OTU	ACE	CHAO	SHANNON	SIMPSON	Phylogenetic Diversity
NOR	292.14 ± 30.93 ^a^	316.85 ± 32.55 ^a^	303.01 ± 31.73 ^a^	2.06 ± 0.16 ^bc^	0.22 ± 0.04 ^ab^	285.29 ± 20.97 ^a^
DSS	304.57 ± 41.04 ^a^	321.36 ± 42.78 ^a^	310.80 ± 41.59 ^a^	2.35 ± 0.26 ^a^	0.18 ± 0.06 ^b^	254.57 ± 41.56 ^ab^
SAL	200.00 ± 85.24 ^b^	225.02 ± 80.78 ^b^	209.94 ± 83.12 ^b^	1.91 ± 0.15 ^c^	0.26 ± 0.04 ^a^	222.43 ± 69.05 ^b^
MES	268.29 ± 46.13 ^a^	290.75 ± 42.59 ^a^	277.57 ± 43.95 ^a^	2.01 ± 0.18 ^c^	0.27 ± 0.07 ^a^	282.43 ± 18.88 ^a^
GCJ	313.57 ± 53.54 ^a^	334.62 ± 53.11 ^a^	321.00 ± 52.90 ^a^	2.24 ± 0.19 ^ab^	0.20 ± 0.06 ^b^	284.86 ± 42.51 ^a^

Data are expressed as the mean ± SD (*n* = 7 per group). Values with different superscripts (a, b, c) are significantly different among groups according to ANOVA with Duncan’s multiple range test at *p* < 0.05.

**Table 4:** Effects of *Gochujang* on relative abundances of gut microbiota at species level in DSS-treated rats.

Species	Composition (%)				
	NOR	DSS	SAL	MES	GCJ
*Enterococcus faecalis*	1.57 ± 0.53 ^bc^	8.37 ± 5.91 ^a^	4.79 ± 2.31 ^b^	2.66 ± 2.13 ^bc^	1.15 ± 0.59 ^c^
*Staphylococcus sciuri* group	1.61 ± 1.38 ^b^	15.34 ± 11.69 ^a^	5.69 ± 4.25 ^b^	0.82 ± 0.80 ^b^	3.02 ± 3.03 ^b^
*Akkermansia muciniphila*	0.04 ± 0.04 ^b^	0.04 ± 0.04 ^b^	0.08 ± 0.08 ^b^	0.17 ± 0.10 ^ab^	0.25 ± 0.26 ^a^
*Enterococcus faecium* group	25.75 ± 6.88 ^bc^	21.51 ± 6.88 ^c^	42.59 ± 9.36 ^a^	38.30 ± 19.89 ^ab^	32.61 ± 13.56 ^abc^
*Escherichia coli* group	26.58 ± 11.94	18.54 ± 12.16	17.61 ± 12.66	22.65 ± 15.46	15.16 ± 7.62
*Lactobacillus reuteri* group	0.06 ± 0.06	0.01 ± 0.01	0.00 ± 0.00	0.01 ± 0.01	0.14 ± 0.28

Data are expressed as the mean ± SD (*n* = 7 per group). Values with different superscripts (a, b, c) are significantly different among groups according to ANOVA with Duncan’s multiple range test at *p* < 0.05.

## Data Availability

The data presented in this study are available upon request to the corresponding author.

## Supplementary Materials

The following are available online at https://www.mdpi.com/article/10.3390/foods10051072/s1, Figure S1: Scheme of the animal study.
