# Supplementary figures and images for: Protective Effect of Gochujang on Inflammation in a DSS-Induced Colitis Rat Model
Source: Foods. 2021 May 12;10(5):1072. doi: 10.3390/foods10051072 (PMC8150376; doi:10.3390/foods10051072)

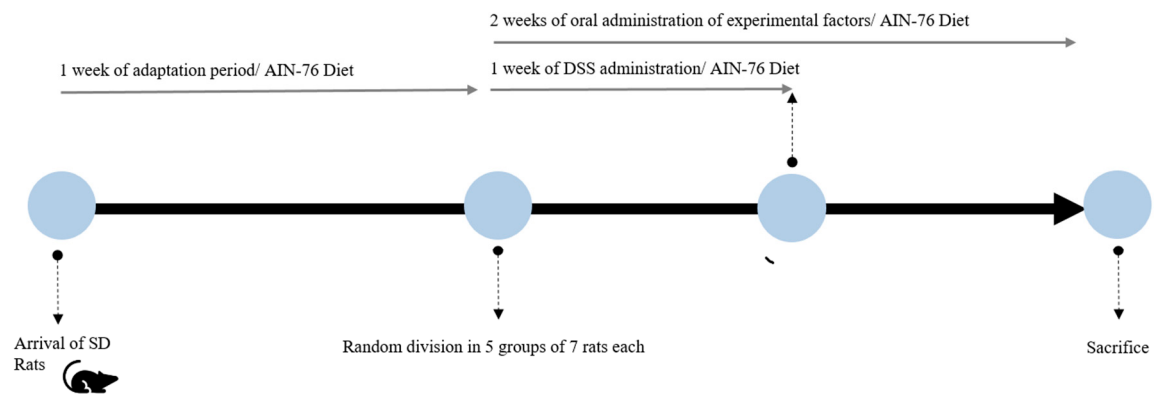

Figure S1. Scheme of the animal study.

Supplement: Supplementary file 1 [file foods-10-01072-s001.zip › foods-1177034-supplementary.pdf]
